# LPS-Induced Systemic Inflammation Caused mPOA-FSH/LH Disturbance and Impaired Testicular Function

**DOI:** 10.3389/fendo.2022.886085

**Published:** 2022-06-23

**Authors:** Peilei Shen, Shuqin Ji, Xulin Li, Qingning Yang, Bingxian Xu, Chris Kong Chu Wong, Liping Wang, Lei Li

**Affiliations:** ^1^ Shenzhen Institute of Advanced Technology, Chinese Academy of Sciences, Shenzhen, China; ^2^ University of Chinese Academy of Sciences, Beijing, China; ^3^ Department of Molecular Biosciences, Northwestern University, Evanston, IL, United States; ^4^ Croucher Institute for Environmental Sciences, Department of Biology, Hong Kong Baptist University Hong Kong, Hong Kong SAR, China; ^5^ Guangdong Provincial Key Laboratory of Brain Connectome and Behavior, Shenzhen, China; ^6^ CAS Key Laboratory of Brain Connectome and Manipulation, Shenzhen, China; ^7^ Shenzhen-Hong Kong Institute of Brain Science–Shenzhen Fundamental Research Institutions, Shenzhen, China

**Keywords:** LPS, HPG axis testis, spermatogenesis, steroidogenesis, BTB, anxiety

## Abstract

Male reproductive function is key to the continuation of species and is under sophisticated regulation, challenged by various stressors including inflammation. In the lipopolysaccharide (LPS) intraperitoneal injection-induced acute systemic inflammation, male fecundity was compromised with decreased testosterone level, damaged spermatogenesis, and downregulations of testicular gene expression levels involved in steroidogenesis regulation and blood–testis barrier. It is also noteworthy that the testis is more sensitive to acute stress caused by LPS-induced systemic inflammation. LPS treatment resulted in lower testicular gene expression levels of steroidogenic acute regulatory protein, cholesterol side-chain cleavage enzyme, and cytochrome P450 family 11 subfamily B member 1 after LPS treatment, while no such decrease was found in the adrenal gland. In parallel to the significant decreases in testicular intercellular adhesion molecule 1, tight junction protein 1, and gap junction alpha-1 protein gene expression with LPS treatment, no decrease was found in the epididymis. In the brain, LPS treatment caused higher medial preoptic area (mPOA) activation in the hypothalamus, which is accompanied by elevated blood follicle-stimulating hormone (FSH) and luteinizing hormone (LH) levels, suggesting a disturbed hypothalamic–pituitary–gonad axis function. Besides mPOA, brain c-fos mapping and quantitative analysis demonstrated a broad activation of brain nuclei by LPS, including the anterior cingulate cortex, lateral septum, paraventricular nucleus of the hypothalamus, basolateral amygdala, ventral tegmental area, lateral habenular nucleus, locus coeruleus, Barrington’s nucleus, and the nucleus of the solitary tract, accompanied by abnormal animal behavior. Our data showed that LPS-induced inflammation caused not only local testicular damage but also a systemic disturbance at the brain–testis axis level.

## Introduction

Male infertility affects about half of couples who cannot conceive (1). The main causes of male infertility can be generally divided into three categories: hypogonadism due to hypothalamic–pituitary abnormity, seminal outflow disability, and testicular dysfunction, while the last one makes up the most common cause of male infertility (1–3). Many kinds of stressors impact normal testes functions, including heat, oxidative stress, environmental contamination, and psychological stress (4–6). Many studies have focused on the cellular and molecular pathways of these stressors on the testes, although the application of most stressors was not restricted to the testes alone and did not solely exert their influence locally at the testes, such as persistent organic pollutants (7, 8) and systemic inflammation (9, 10). Testicular dysfunctions were noticed in plenty of pathological states of systemic inflammation (10–12), while much less attention was paid to the contribution of systemic inflammation to testicular dysfunctions. On the other hand, while the testicular function is under sophisticated regulation of the central nervous system (CNS) through the classical hypothalamic–pituitary–gonadal (HPG) axis, most studies have focused on testicular function regulation at the peripheral organ level (13); there is limited evidence of possible CNS participation in stress-induced testes dysfunction, which is perhaps surprising considering the well-established role of HPG axis in regulating testicular functions.

Clinically, close relationships between male reproductive diseases such as male infertility (14, 15), orchialgia (16), and psychological stress, anxiety, or depression have been reported. A study of patients undergoing *in vitro* fertilization showed that both state and trait anxiety had a negative impact on ejection volume, sperm count, and sperm motility (17). The negative association between semen quality and perceived stress has also been observed (18, 19). Infertile men reported a higher number of stressful life events compared to a fertile group (20). In addition, psychological stress such as earthquake experience was shown to significantly impair the semen quality of survivors even after many years, demonstrating long-term effects (21, 22). Despite the reported association between testis-related diseases and psychological disorders, evidence addressing the underlying mechanisms of the systemic regulation at both the central and peripheral levels in animal models is scarce.

Lipopolysaccharide (LPS) was reported to induce brain inflammation and disturbance of the blood–testis barrier (BTB) as well (23, 24). In this study, we applied LPS, which is an important constituent of the outer membrane of Gram-negative bacteria to induce orchitis (25) and systemic inflammation as an acute stressor. Here, we proposed the hypothesis that CNS is involved in the progress of testicular dysfunction at acute systemic inflammation stress. The impact of LPS administration on testosterone production, the integrity of BTB, spermatogenesis, brain nuclei activation, and corresponding behavior outputs were studied. Our study offers an approach to study possible CNS involvement in acute stress-induced testicular impairment.

## Materials and Methods

### Animals and LPS treatment

Adult (6–8 weeks) C57BL/6J male mice (Beijing Vital River Laboratory Animal Technology Co., Ltd., Beijing, China) were housed in groups of five, had ad libitum access to food and water and were maintained on a 12:12-h light/dark cycle (lights on from 8:00 a.m. to 8:00 p.m.). Mice were given a single dose of 4 mg/kg lipopolysaccharides (LPS, L2630, Sigma, Saint Louis, Missouri, USA) or same volume of saline *via* intraperitoneal (i.p.) injections. All husbandry and experimental procedures in this study were approved by the Animal Care and Use Committees of the Shenzhen Institute of Advanced Technology (SIAT), Chinese Academy of Sciences (CAS), China.

### Radio-Immunoassay Assay of Testosterone

Mice plasma samples were collected at the afternoon of the experimental day. Plasma testosterone levels were measured using an Iodine[^125^I] Radio-Immunoassay Assay (KIP1709, DIasource, Louvain-la-Neuve, Belgium). Blood samples from the LPS-treated and control mice were collected with ethylenediaminetetraacetic acid (EDTA)-pretreated centrifuge tubes and gently mixed at room temperature before centrifugation to prevent blood coagulation. Supernatant plasma was collected and stored at −80°C prior to use. Standard testosterone samples were used to prepare a standard curve. Both standard testosterone samples and plasma samples were incubated with rabbit anti-testosterone antibody at 37°C for 1 h. Then, donkey anti-rabbit separating agent was added, and the mixture was kept still at room temperature for another 15 min prior to centrifugation at 3,500×*g* for 10 min. The absorbance was normalized to standard testosterone to calculate each sample (Beijing North Institute of Biotechnology Co., Ltd., Beijing, China).

### MILLIPLEX^®^ Multiplex Assays Using Luminex^®^ Testing Plasma Cytokine Concentration

Plasma samples were analyzed with Endocrine Multiplex Assay (MADKMAG-71K, Merck Millipore, Burlington, Massachusetts, USA) to determine levels of interleukin 6 (IL6), tumor necrosis factor alpha (TNFα), monocyte chemoattractant protein 1 (MCP1), according to the manufacturer’s protocol. Briefly, after incubation of the assay buffer for 10 min at room temperature, experimental plasma samples or gradient dilution standard samples were added to the wells of a 96-well plate and supplemented with assay buffer, serum matrix, and then neat samples. Dyed antibody-bound beads were then added and shaken gently before being left to incubate overnight at 4°C. Next, detection antibodies were added to each well following a washing step and incubated with agitation for 30 min at room temperature. Streptavidin–phycoerythrin was added to the plate and was incubated for another 30 min. The plate was washed for three times, and sheath fluid was added to resuspend the beads for 5 min. Fluorescent intensity quantification was performed using a Luminex analyzer (Luminex 200, Merck Millipore, Burlington, Massachusetts, USA). Data were analyzed using MILLIPLEX software (Analyst.V5.1, Merck Millipore, Burlington, Massachusetts, USA).

### Enzyme-Linked Immunosorbent Assay of Luteinizing Hormone and Follicle-Stimulating Hormone

Plasma luteinizing hormone (LH) and follicle-stimulating hormone (FSH) were measured using ELISA kit (EM1188 and EM1035, FineTest, Wuhan, China) according to the manufacturer’s instructions. Briefly, experimental plasma samples and gradient dilution standard samples were incubated with biotin-labeled antibody in the pre-coated plate, respectively, for 45 min at 37°C. The plates were washed with wash buffer for three times; then, the horseradish peroxidase –streptavidin conjugate was added to the plates and incubated for 30 min at 37°C. The plates were washed five times with wash buffer; TMB substrate was added and incubated at 37°C in dark for 15 min. When the standard wells showed apparent gradient, stop solution was added to each well and subjected to a 450 nm in a microplate reader (Synergy H1, Bio-Tek, Santa Clara, California, USA) immediately. The absorbance was normalized to standard LH and FSH, respectively, to calculate the value for each sample.

### Hematoxylin and Eosin Histology and c-fos Immunofluorescence Staining

Forty-eight hours after LPS injection, mice were put under deep anesthesia and transcardially perfused 4% paraformaldehyde (PFA) in phosphate-buffered saline (PBS), using saline-injected mice as controls. Histological analysis of H&E and immunofluorescence staining was performed as previously reported (26). For H&E staining, fixed testes and epididymis were harvested and post-fixed with 4% PFA for 24 h, then embedded in paraffin, sliced into 4-μm sections with a microtome (RM2016, Lecia, Wetzlar, Germany), and subjected to deparaffinization, hydration, and then stained with hematoxylin and eosin (AS1055A and AS1094, Aspen, Wuhan, China). For c-fos mapping, the brains were harvested, post-fixed with 4% PFA, and cryoprotected with 30% sucrose in PBS. For immunofluorescence staining, mouse brains were dehydrated in 30% sucrose and then sliced into 30-µm sections using cryostat (CM1860, Lecia, Wetzlar, Germany). Brain sections were stored at −20°C with a cryoprotectant prior to histological analyses. Tissue sections were first washed with PBS to remove embedding medium; antibody staining was performed in 24-well plates on floating tissue sections. Sections were blocked with normal goat serum for 1 h at room temperature and then incubated overnight in primary antibodies at 4°C, followed by PBS washing and incubation with secondary antibodies at room temperature for 2 h. The primary antibodies used in this study were rabbit anti-c-fos (2250, Cell Signaling Technology, Danvers, Massachusetts,USA, 1:500). Alexa Fluor@488-conjugated AffiniPure fab fragment goat anti-rabbit secondary antibody (111-547-003, Jackson ImmunoResearch, Philadelphia, Pennsylvania, USA, 1:200) was used. Sections were then incubated with 4′,6-diamidine-2′-phenylindole dihydrochloride (DAPI, D9542, Sigma, Saint Louis, Missouri, USA, 0.4 mg/ml) for 5 min. Confocal images were taken with a confocal microscope (LSM 880, Zeiss, Oberkochen, Germany) and scanning images were taken with a virtual microscopy slide scanning system (VS120, Olympus, Tokyo, Japan).

### RNA Extraction and Real-Time Quantitative PCR

To explore expression levels of blood–testis–barrier-related, steroidgenesis and inflammatory genes, testicular tissues, epididymis tissues, adrenal gland and hypothalamus tissues from LPS-treated and control animals were collected and digested to perform real-time quantitative PCR analysis. Total RNA was extracted from the testis, epididymis tissue or hypothalamus tissue with Trizol reagent (15596018, Invitrogen, Carlsbad, California, USA) and quantified using a micro-spectrophotometer (NanoDrop One, Thermo Scientific, Massachusetts, USA). One microgram of total RNA was used for cDNA synthesis using ReverTra Ace qPCR RT Kit (FSQ-101, TOYOBO, Osaka, Japan). Real-time quantitative PCR was performed using SYBR^®^ Green Real-Time PCR Master Mix (QPK-201, TOYOBO, Osaka, Japan), with a LightCycler^®^ 480 Instrument (Roche, Basel, Switzerland). β-Actin was used to normalize expression levels of the genes tested. The primers used in this study are shown in **Table 1**.

**Table 1:** Primers sequences used in the real-time quantitative PCR reaction.

Transcripts	Forward primer	Reverse primer
*β-actin*	GGCTGTATTCCCCTCCATCG	CCAGTTGGTAACAATGCCATGT
*Icam-1*	TTCCAGCTACCATCCCAAAG	AGCTTCAGAGGCAGGAAACA
*Tjp1*	ACTCCCACTTCCCCAAAAAC	CCACAGCTGAAGGACTCACA
*Gja1*	TACCACGCCACCACCGGCCCA	GGCATTTTGGCTGTCGTCAGGGAA
*StaR*	ATGTTCCTCGCTACGTTCAAG	CCCAGTGCTCTCCAGTTGAG
*P450scc*	AGGTCCTTCAATGAGATCCCTT	TCCCTGTAAATGGGGCCATAC
*3β-HSD*	TATTCTCGGTTGTACGGGCAA	GTGCTACCTGTCAGTGTGACC
*Cyp11b1*	ATAGAAGCTAGCCACTTTGT	AGGGTGTGGAGGAACTTCAG
*Il1β*	CTCGCAGCAGCACATCAACAA	AAGGTCCACGGGAAAGACACA
*Il6*	TAGTCCTTCCTACCCCAATTTCC	TTGGTCCTTAGCCACTCCTTC
*Tnfα*	CAGGCGGTGCCTATGTCTC	CGATCACCCCGAAGTTCAGTAG
*Tlr2*	TGTGCCACCATTTCCACG	AAAGGGCGGGTCAGAGTT
*Tlr4*	ATGGCATGGCTTACACCACC	GAGGCCAATTTTGTCTCCACA

### Animal Behavior Test and Analysis

An open-field test (OFT) was used to assess anxiety-like behavior and locomotor activity in an open-field arena (50 cm × 50 cm × 50 cm). For analysis, the center of the arena floor covering 25% of the total floor area was defined as the center area (i.e., 25 cm × 25 cm). The OFT was used to assess behavior following LPS stress. Mice were first allowed to freely explore the open field for 1–2 min prior to a 10-min observation session. The number of entries to the center area and time spent in the center during the 10-min session were recorded and analyzed using Anymaze software (Stoelting, Chicago, Illinois, USA). For elevated plus maze (EPM) test, mice were put on a four-armed plus maze, at the center of two open and two closed arms (white PVC, 30 cm length × 5 cm width per arm) raised 50 cm above the floor, for 10-min sessions. The OFT and EPM were cleaned between mice with 20% ethanol solution. The number of entries to the open arms and time spent in the open arms were recorded and analyzed by Anymaze software (Stoelting, Chicago, Illinois, USA). To access the effect of low dose of LPS treatment on animal behaviors, an extra group of mice were treated with 0.4 mg/kg LPS for 48 h, besides the 4 mg/kg LPS treatment.

### Statistical Analysis

All data in this study were analyzed as mean ± SEM. Statistical significance was calculated using GraphPad Prism (GraphPad Software, San Diego, California, USA) and labeled as ns, p > 0.05, *p < 0.05, **p < 0.01, ***p < 0.001, and ****p < 0.0001. Student’s t-test or Kruskal–Wallis with Dunn *post-hoc* test was used as appropriate. All n values in this study refer to the number of mice used in each experiment, as shown in each figure legend.

## Results

### LPS Treatment Resulted in Elevated Circulating Inflammatory Cytokines

**Figure 1:**
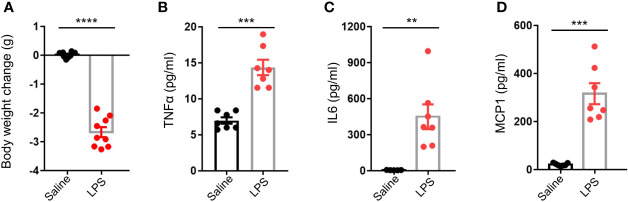
LPS treatment resulted in an elevation of circulating inflammatory cytokines. **(A)** LPS treatment resulted in lower mice body weight (data are presented as mean ± SEM, n = 9 saline, n = 9 LPS, Student’s t-test, **** p < 0.0001). **(B–D)** LPS treatment resulted in higher plasma levels of tumor necrosis factor alpha (TNFα), interleukin 6 (IL6), and monocyte chemoattractant protein 1 (MCP1) compared to controls (data are presented as mean ± SEM, n = 5–7 saline, n = 7 LPS; Student’s t-test, ***p < 0.001, **p < 0.01).

Adult male C57BL/6J mice were intraperitoneally injected with a single dose of either 4 mg/kg LPS in 0.9% saline or with 0.9% saline only. After 48 h, bodyweight decrease in the LPS group was significantly larger than that of the control group (p < 0.0001, **Figure 1A**). There were higher plasma levels of inflammatory cytokines such as TNFα (p < 0.001), IL6 (p < 0.01), and MCP1 (p < 0.001) in the LPS group compared to the controls (**Figures 1B–D**), gene expression analysis of testis also revealed local testicular inflammation (**Supplementary Figure 1**). In summary, acute stress caused by LPS administration resulted in a significant physiological disturbance in terms of inflammation.

### LPS Treatment Impaired Spermatogenesis and Decreased Gene Expression of Blood–Testis–Barrier-Related Proteins

**Figure 2:**
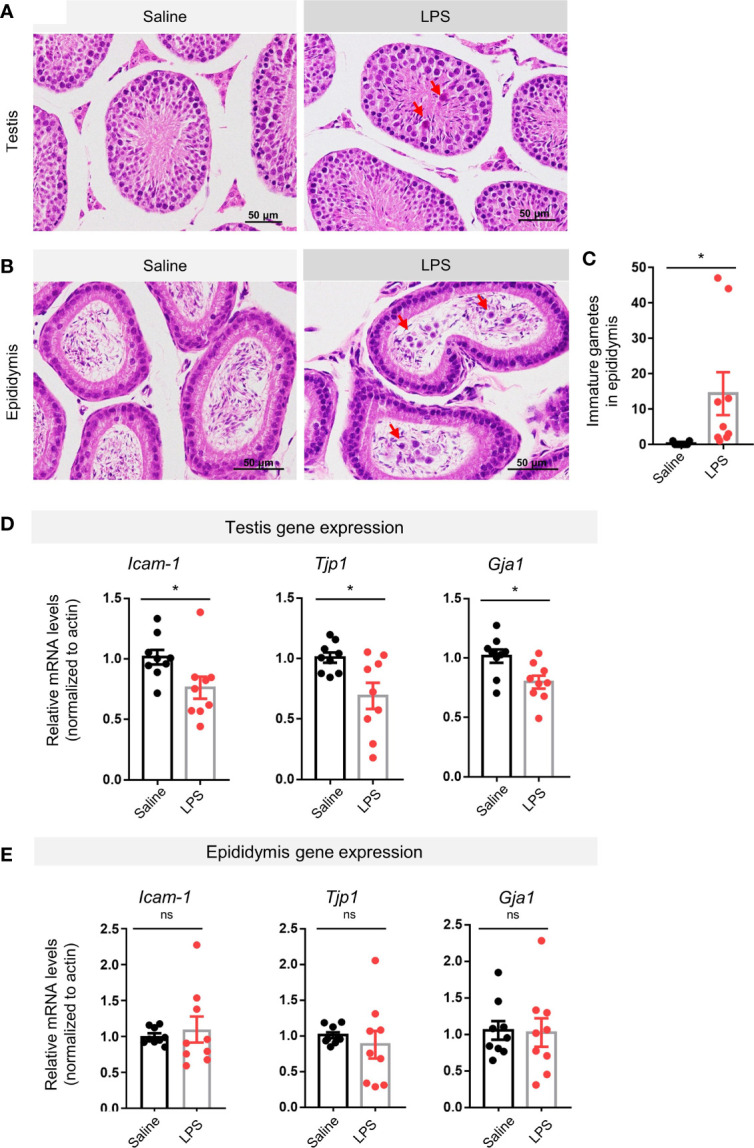
LPS treatment resulted in impaired spermatogenesis and disrupted blood–testis barrier-related genes expression. **(A)** LPS-treated mice showed immature germ cells dropped from the testicular wall (H&E staining, scale bars = 50μm). **(B, C)** LPS-treated mice showed that immature germ cells accumulated in the lumen of epididymis, which were quantitatively different to control in cell count numbers (H&E staining, scale bars = 50 μm; data are presented as mean ± SEM, n = 9 saline, n = 9 LPS; Student’s t-test, *p < 0.05). **(D, E)** The LPS-treated group had lower gene expression levels of intercellular adhesion molecule 1 (*Icam-1*), tight junction protein 1 (*Tjp1*), and gap junction alpha-1 protein (*Gja1*) than controls, while no such changes were observed in the epididymis of the LPS-treated mice (data are presented as mean ± SEM, n = 9 saline, n = 9 LPS, *p < 0.05, ns, p > 0.05).

H&E staining revealed immature male gametes in the lumen of seminiferous tubules of the testis (**Figure 2A**) and in the lumen of the epididymis (**Figure 2B**) 48 h after LPS treatment. Quantification of the gametes in the epididymis showed a statistical difference between the LPS treatment group and the saline control group (p < 0.05, **Figure 2C**). The BTB lays the structural foundation of the spermatogenesis niche (27). Real-time quantitative PCR analysis of the testicular tissue revealed lower expression levels of genes coding for BTB-related proteins, including intercellular adhesion molecule 1 (*Icam-1*, p < 0.05), tight junction protein 1 (*Tjp1*, p < 0.05), and gap junction alpha-1 protein (*Gja1*, p < 0.05) in the LPS-treated groups compared to controls (**Figure 2D**). Using the epididymis tissue as a control, we found no such difference in gene expression levels of *Icam-1*, *Tjp1*, or *Gja1* in the LPS-treated epididymis, indicating that LPS administration led to a dysfunction of cell–cell interaction in testes, rather than the epididymis (**Figure 2E**).

### The LPS-Treated Group Had Lower Plasma Testosterone Levels and Suppressed Gene Expression of Key Steroidogenesis Enzymes

**Figure 3:**
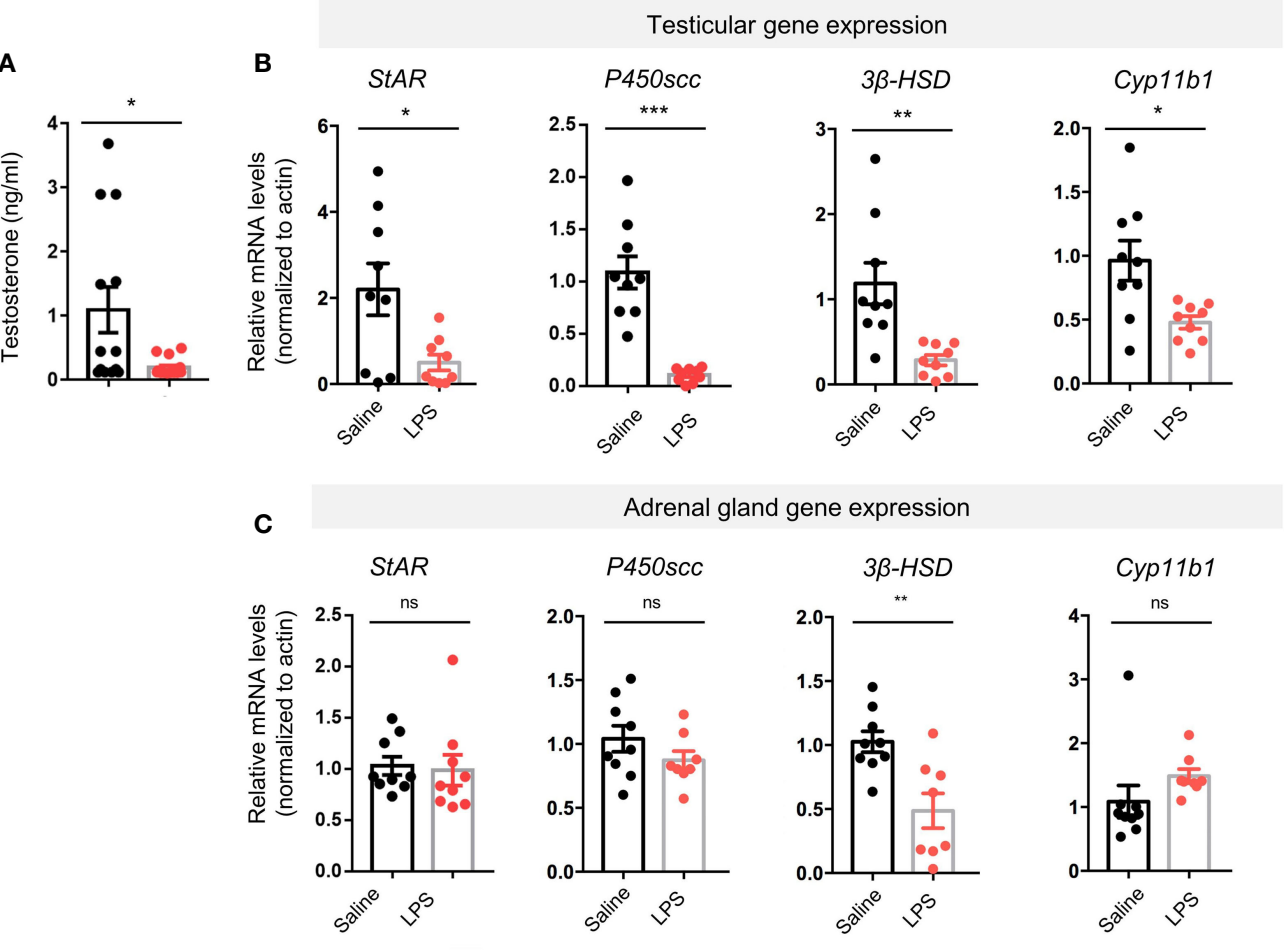
LPS treatment resulted in decreased levels of plasma testosterone and testicular steroidogenesis-regulating proteins gene expression. **(A)** The LPS-treated group had lower plasma testosterone levels compared with saline controls (data are presented as mean ± SEM, n = 13 saline, n = 16 LPS; Student’s t-test, *p < 0.05). **(B, C)** Real-time quantitative PCR results showed LPS treatment resulted in lower gene expression levels of steroidogenic acute regulatory protein (*StAR*), cholesterol side-chain cleavage enzyme (*P450scc*), 3β-hydroxysteroid dehydrogenase (*3β-HSD*), and cytochrome P450 family 11 subfamily B member 1 (*Cyp11b1*) compared with controls. In adrenal glands of the LPS-treated mice, no significant changes in the steroidogenesis-regulating proteins gene expression were observed except for *3β-HSD* (data are presented as mean ± SEM, n = 9 saline, n=8-9 LPS, Student’s t-test, ***p < 0.001, **p < 0.01, *p < 0.05, ns, p > 0.05).

Radioimmune assay (RIA) showed that LPS-treated group had lower plasma testosterone levels than the controls (p < 0.05, **Figure 3A**). We then performed real-time quantitative PCR to evaluate gene expression levels of key steroidogenesis enzymes using another batch of mice. Reduction in steroidogenic acute regulatory protein (*StAR*, p < 0.05), cholesterol side-chain cleavage enzyme (*P450scc*, p < 0.001), 3β-hydroxysteroid dehydrogenase (*3β-HSD*, p < 0.01), and cytochrome P450 family 11 subfamily B member 1 (*Cyp11b1*, p < 0.05) was found in the LPS treatment group compared to controls (**Figure 3B**). In the adrenal glands, another primary steroidogenesis organ, only one steroidogenic gene had reduced expression (*3β-HSD*, p < 0.01), whereas expression of the other three genes were not different between groups (*StAR*, p = 0.8129; *P450sc*c, p = 0.1979; *Cyp11b1*, p = 0.1740; **Figure 3C**). These results suggest that LPS treatment caused an impairment in male sex hormone production. The testes seem to be more vulnerable to the LPS-caused stress than the adrenal glands in terms of steroidogenic gene expression levels.

### LPS Treatment Activated the Medial Preoptic Area in the Hypothalamus and Increased Circulating Follicle-Stimulating Hormone and Luteinizing Hormone

**Figure 4:**
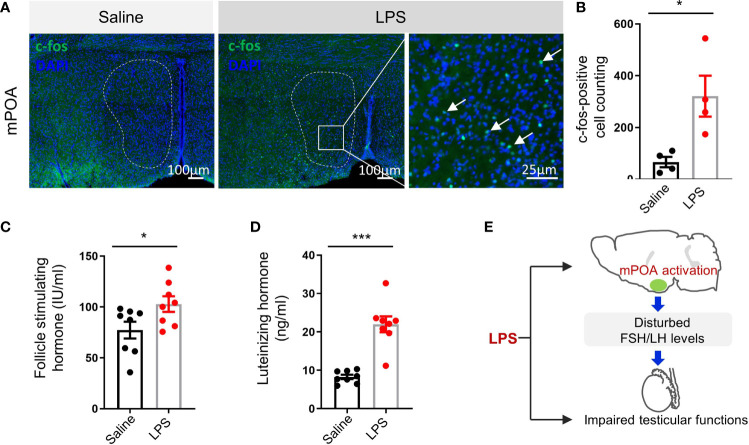
LPS treatment resulted in the activation of medial preoptic area (mPOA) in the hypothalamus and an increase in circulating follicle-stimulating hormone (FSH) and luteinizing hormone (LH) levels. **(A)** Representative images of c-fos immunostaining in medial preoptic area (mPOA, green, c-fos; blue, DAPI; scale bars = 100 and 25 μm). **(B)** Quantitative analysis of c-fos positive neurons in the mPOA activated by LPS treatment (data are presented as mean ± SEM, n = 4 saline, n = 4 LPS; Student’s t-test, *p < 0.05). **(C, D)** The LPS-treated group had higher plasma FSH and LH levels compared with controls (data are presented as mean ± SEM, n = 8 saline, n = 8 LPS; Student’s t-test, ***p < 0.001, *p < 0.05). **(E)** A summary graph of disturbed mPOA-FSH/LH axis by LPS-induced systemic inflammation.

We observed abnormal behaviors after LPS treatment. When subjected to OFT, LPS-treated mice showed decreases in total distance travelled, entries to center, and time in center (**Supplementary Figures 2A, B**). In the EPM test, LPS treatment also induced decrease in total distance travelled and a trend in Entries to Open arms and Time in Open arms (**Supplementary Figures 2C, D**). To clarify the behavioral output of the LPS treatment, controls, 0.4 mg/kg LPS-treated group, and 4 mg/kg LPS-treated group were evaluated with OFT and EPM. The lower dose of LPS treatment caused no significant difference in locomotion and an increase in anxiety-like behavior (**Supplementary Figures 2E–G**). The abnormal behavior caused by LPS treatment suggested neurotoxicity of LPS as reported before. Gene expression analysis of hypothalamic tissues revealed elevated levels of inflammatory cytokines (**Supplementary Figure 3**). Considering the importance of the HPG axis in regulating testicular function (28), we wanted to know whether LPS treatment induced any change in neuronal activity in the hypothalamus, especially the medial preoptic area (mPOA) where most the gonadotropin-releasing hormone (GnRH) neurons are located. Immunostaining of c-fos early response gene as an indicator of neuronal activation showed significant increase in the mPOA of the LPS group (**Figures 4A, B** p < 0.05). ELISA results showed changes in circulating FSH (p < 0.05) and LH (p < 0.001) levels after LPS treatment (**Figures 4C, D**), suggesting LPS-treatment resulted in disturbed HPG-axis functions (**Figure 4E**).

### LPS-Treatment Resulted in Vast Brain Activation

**Figure 5:**
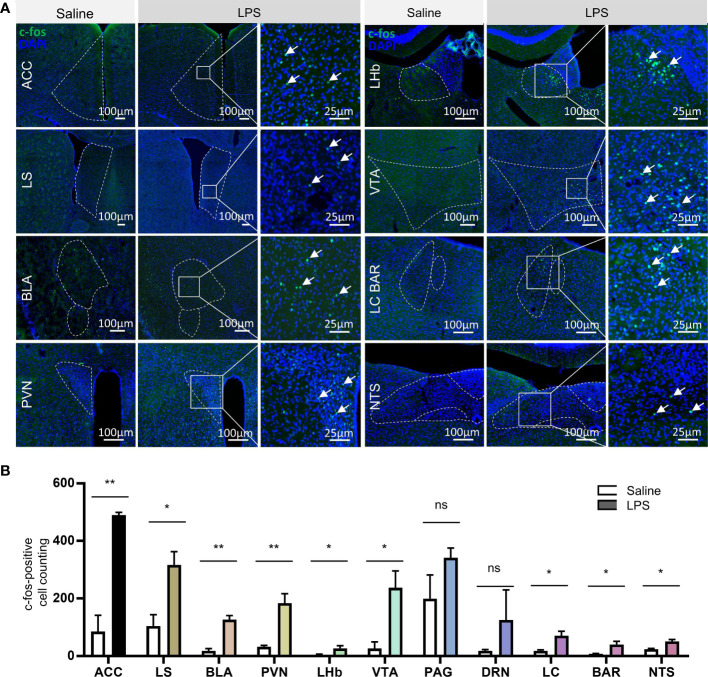
LPS treatment caused broad brain nuclei activation related to stress response. **(A)** Representative images of c-fos immunostaining in brain nuclei, including the anterior cingulate cortex (ACC), lateral septum (LS), basolateral amygdala (BLA), paraventricular nucleus of the hypothalamus (PVN), lateral habenular nucleus (LHb), ventral tegmental area (VTA), locus coeruleus (LC), Barrington’s nucleus (BAR), and nucleus of the solitary tract (NTS) (green, c-fos; blue, DAPI; scale bars = 100 and 25 μm). **(B)** Quantitative analysis of the c-fos-positive neurons in the above brain nuclei showed significant increase in neural activation in nuclei including ACC, LS, BLA, PVN, LHb, VTA, LC, BAR, and NTS, which are all stress response nuclei (data are presented as mean ± SEM, Student’s t-test, n = 3-4 for saline, n = 3–4 for LPS, **p < 0.01; *p < 0.05 ns, p > 0.05).

Besides mPOA, c-fos immunofluorescent staining showed broad activation of brain nuclei in the LPS-treatment group than controls, including nuclei of the forebrain, thalamus, hypothalamus, brainstem, and cortex. Cell counting and analysis showed that there was significantly higher number of c-fos-positive cells in the LPS group compared to the saline control group in the following brain nuclei: the anterior cingulate cortex (ACC), lateral septum (LS), basolateral amygdala (BLA), paraventricular nucleus of the hypothalamus (PVN), lateral habenular nucleus (LHb), ventral tegmental area (VTA), locus coeruleus (LC), Barrington’s nucleus (BAR), and the nucleus of the solitary tract (NTS) (**Figures 5A, B**). Interestingly, most of the LPS-activated brain nuclei that we observed are participants in anxiety circuits and involved in stress responses (29), which was in line with the behavioral outputs that we observed (**Supplementary Figure 2E–G**).

## Discussion

Our study demonstrates that testis impairment, including a reduction in testosterone level and impaired spermatogenesis, can be induced by acute LPS stress. These changes were accompanied by a significant reduction in BTB-related gene expression, decreased steroidogenesis regulating protein gene expression, activation of mPOA, disturbed HPG-axis function, activation of stress-related brain nuclei, and, furthermore, anxiety-like behavior.

LPS intraperitoneal (i.p.) injection had been used to induce atypical orchitis model in rodents (30, 31), and we observed elevated testicular gene expression of inflammatory cytokines (**Supplementary Figure 1**). LPS-induced systemic inflammation resulted in damaged spermatogenesis and testicular steroidogenesis. Yet in the previous reports, the underlying mechanism was focused on the local testicular mechanism. As a matter of fact, LPS injection caused a systemic inflammation in the whole body instead of local testicular inflammation, as we found that LPS indeed induced a systemic inflammation with elevated levels of IL6, TNFα, and MCP1. Importantly, in clinical reports, systemic inflammation is more common than inflammation that happens only inside the testis (10–12). It would be intriguing to find that LPS-caused systemic inflammation would affect testicular function through the hypothalamus–pituitary–gonad axis.

Leydig and Sertoli cells are very important for testicular functions, and they have been intensively studied by previous works using *in vivo* and *in vitro* experiments with LPS exposure (32–34). In our study, we accessed the testosterone levels between the control and LPS-treated groups, as a direct indicator of Leydig cell function. Furthermore, in line with a previous report (32), LPS treatment caused a decrease in plasma testosterone levels, which was accompanied by decreased gene expression levels of steroidogenesis-regulating enzymes including *StAR*, *P450scc*, *3β-HSD*, and *Cyp11b1.* It is interesting to notice that, using adrenal gland as a control organ, gene expression levels of *P450scc*, *3β-HSD*, and *StAR* in the adrenal gland were not so badly impaired as in the testes. The testes seem to be more vulnerable to the acute stress caused by LPS injection than the adrenal glands in terms of steroidogenic gene expression levels.

Besides the damaged hormone-producing function of testis, our results also showed that LPS injection caused impaired spermatogenesis, as demonstrated by immature gametes in the lumen of testicular tubules and epididymis lumen. This impaired spermatogenesis might be caused by disturbed BTB as previously proposed (27, 35, 36), as weakened BTB negatively affects different stages of spermatogenesis (37). For example, *Icam-1* downregulation is involved in BTB disturbance and spermatogenesis impairment in busulfan-treated mice (38). The junctions between Sertoli cells of the seminiferous tubules, near the base membrane (39), are the most important component of BTB. Sertoli cells were reported to have high expression levels of *Icam-1*, *Tjp1*, and *Gja1* (40, 41). BTB-related ZO-1/TJP1 protein level can be downregulated by heat stress (42); direct TNFα administration reduces expression of the *Tjp1* in the testes and disrupts the BTB (43). Homozygous knockout of *Gja1*, another BTB gene that codes the gap junction protein connexin 43, leads to infertility in mice (44). In our study, we found a significant decrease in gene expression levels of *Icam-1*, *Tjp1* or *Gja1* in the testis, while no such decrease in BTB-related gene expression was observed in the epididymis. In another word, in LPS-induced epididymitis, BTB-related gene expression was not as badly affected as in the LPS-induced orchitis, indicating a different regulatory mechanism under stress between the two reproductive organs, which again suggests the vulnerability of the testes as a vital reproductive organ.

Furthermore, LPS administration has been reported to induce brain inflammation (45, 46), impair neurogenesis and memory (47), and even cause sporadic Alzheimer’s disease (48). The receptor of LPS, TLR4, was reported to be induced in the brain by LPS treatment. There was elevated expression of TLR4 in both glia and neuron, with wide expression in microglia (49), astrocyte (50), oligodendrocyte (49), and neuron (51). A previous report showed that LPS injection induced a significant increase in LH at about 2.5 days after LPS treatment (52). Another study indicated a time-dependent increase in LH and FSH after LPS engagement (53). Considering the important regulations of the HPG axis on testicular functions, we studied the mPOA of the hypothalamus, where most GnRH neurons are located, and is key to the HPG-axis regulation. Our results showed an activation of the mPOA brain nucleus by LPS injection, together with elevated circulating levels of LH and FSH. Hence, LPS treatment induced a stressed state not only in the testes but a broader disturbance including the brain. We speculated that this elevated LH and FSH levels observed in our study could be a direct result from mPOA activation. It could also be the secondary response of disinhibition of the HPG axis following the decreased testosterone production from the testis. Lower testosterone due to the impairment of steroidogenesis led to subsequent weakened negative feedback on the CNS. This disturbance of HPG-axis function might in turn contribute to the dampened steroidogenesis, damaged spermatogenesis, and perturbance of BTB, suggesting that LPS-caused acute inflammation not only disturbed testicular function through local testicular mechanism but also through multilevel of HPG axis to impact testicular function.

Interestingly, mPOA was not the only brain nucleus activated by LPS injection; broad brain nuclei including ACC, LS, BLA, PVN, LHb, VTA, LC, BAR, and NTS, were found to be activated, too. Most of the above nuclei are reported to be upstream of mPOA with monosynaptic connection to mPOA (54–56). It has been long known that VTA innervates mPOA through dopaminergic projections (54). Retrograde tracing revealed direct projection from LS, PVN, VTA, LC, amygdala (55), and NTS (56) to mPOA. Among these activated nuclei, the PVN is one of the most interesting, since it plays an important role in stress response through the hypothalamic–pituitary–adrenal (HPA) axis (57) and has direct neural connections to mPOA (58). Another interesting nucleus is the brainstem LC, which is the main source of the brain noradrenergic system and mediates stress responses (59). Indeed, our observation is in accordance with a study showing that LPS treatment induced an elevation in the level of tyrosine hydroxylase mRNA and norepinephrine biosynthesis in the LC (60). Reports from our own group and others have shown that LC noradrenergic neurons are activated by stress, and optogenetic activation of these neurons induces typical anxiety-like behavior (59, 61). In addition, we found that all the brain nuclei activated by LPS all involved in stress-response and anxiety circuit (62–65). Indeed, our behavioral analysis confirmed the anxiety-like behavior in the LPS-treated mice.

According to our results, testis is not the only organ target of LPS-caused systemic inflammation, yet it showed very unique response to the stress compared with adrenal gland in steroidogenesis and compared with epididymis in BTB related proteins. Our study demonstrates that besides local testicular effect, LPS injection caused disturbance in the HPG axis in terms of mPOA activation and gonadotropins production. Yet, one limitation of our current study is that we provide no detailed cell-type-specific neural circuit basis for the mPOA activation, although potential upstream candidates are provided (54–56) and most of the upstream candidates were activated by LPS. In addition, our study does not provide information on the full temporal extent of this LPS-caused pathological state, as we only used two doses of LPS and all the testicular and brain effects were examined in an acute manner. Thus, further investigation using chronic LPS treatment may be useful to examine the long-term mechanism of the brain–testis axis in regulating testicular function and the possible adaptation of chronic treatment over time. Reversible manipulation of responding neurons using optogenetic or chemogenetic tools may provide more direct evidence toward our understanding of CNS involvement. Additionally, it would be useful to determine a complete brain-network mechanism, including cell-type-specific circuits, especially those circuits upstream of the hypothalamic–pituitary–gonad axis and their dynamic changes under stress.

## Data Availability Statement

The original contributions presented in the study are included in the article/**Supplementary Material**. Further inquiries can be directed to the corresponding authors.

## Ethics Statement

The animal study was reviewed and approved by the Animal Care and Use Committees of the Shenzhen Institute of Advanced Technology (SIAT), Chinese Academy of Sciences (CAS), China.

## Author Contributions

LL, LW, and CW conceived and designed the project. PS collected the data. SJ, XL, QY, and BX contributed to data or analysis. LL and PS performed the analysis. PS and LL wrote the paper. All authors contributed to the article and approved the submitted version.

## Funding

This work was supported by the National Natural Science Foundation of China (NSFC, grant numbers NSFC31971072, NSFC32171154, and NSFC31471109); the Key Collaborative Research Program of the Alliance of International Science Organizations (ANSO-CR-KP-2021-12); Guangdong Province Grant (grant numbers 2019A050510032, 2017B030301017, and 2018B030331001); and China Shenzhen Science Technology and Innovative Commission (SZSTI, grant numbers SZSTI JCYJ20180508152336419 and GJHZ20200731095406021).

## Conflict of Interest

The authors declare that the research was conducted in the absence of any commercial or financial relationships that could be construed as a potential conflict of interest.

## Publisher’s Note

All claims expressed in this article are solely those of the authors and do not necessarily represent those of their affiliated organizations, or those of the publisher, the editors and the reviewers. Any product that may be evaluated in this article, or claim that may be made by its manufacturer, is not guaranteed or endorsed by the publisher.

## Supplementary Material

Supplementary Figure 1 LPS caused increase of inflammatory cytokines gene expression in the mouse testis. Relative mRNA levels normalized to actin showed significant increase in the levels of *Tnfα* and *Tlr2* (data are presented as mean ± SEM, n = 3 Saline, n = 4 LPS; Student’s t test, ** p < 0.01, * p < 0.05, ns, p > 0.05).

Supplementary Figure 2 LPS treated mice showed abnormal behaviors in open-field test and elevated plus maze test 48 hours after LPS treatment. **(A)** Example of OFT tracking traces of mice in the saline control and LPS groups. **(B)** LPS-treated mice had decreased total distance travelled, fewer entries to the center and less time in the center (data are presented as mean ± SEM, n = 9 Saline, n = 9 LPS; Student’s t test, ***p < 0.001, *p < 0.05). **(C)** Example of EPM tracking traces of mice in the saline control and LPS groups. **(D)** LPS-treated mice had decreased total distance travelled (data are presented as mean ± SEM, n = 9 Saline, n = 9 LPS; Student’s t test, *p < 0.05, ns, p > 0.05). **(E)** In open-field test, LPS at 4mg/kg caused decreases in Total distance travelled, Entries to center and Time in center, LPS at 0.4mg/kg caused decreases in Total distance travelled, Entries to center and Time in center (data are presented as Violin plot, n = 17 Saline, n = 8 0.4mg/kg LPS, n = 18 4mg/kg LPS; Kruskal-Wallis with Dunn *post-hoc* test, ***p < 0.001, **p < 0.01, *p < 0.05, ns, p > 0.05). **(F–G)** In the elevated plus maze test, 4 mg/kg LPS caused decrease in total distance travelled, 0.4mg/kg LPS caused no significant difference in Total distance travelled and a significant decrease in the ratio of Entries to Open Arms and Entries to Close Arms (data are presented as Violin plot, n = 17 Saline, n = 8, 0.4mg/kg LPS, n = 18 for 4mg/kg LPS; Kruskal-Wallis with Dunn *post-hoc* test, *p < 0.05, ns, p > 0.05).

Supplementary Figure 3 LPS caused increase of inflammatory cytokines gene expression in the mouse hypothalamus. Relative mRNA levels normalized to actin showed significant increase in the levels of *Tnfα*, *IL1β*, *Tlr2* and *Tlr4* (data are presented as mean ± SEM, n = 3 Saline, n = 4 LPS; Student’s t test, ** p < 0.01, * p < 0.05, ns, p > 0.05).

## References

[1] Sharma A Minhas S Dhillo WS Jayasena CN. Male Infertility Due to Testicular Disorders. J Clin Endocrinol Metab (2020) 106(2):e442–e59. doi: 10.1210/clinem/dgaa781 PMC7823320 33295608

[2] Dubin L Amelar RD. Etiologic Factors in 1294 Consecutive Cases of Male Infertility. Fertil Steril (1971) 22(8):469–74. doi: 10.1016/S0015-0282(16)38400-X 4398669

[3] Carlsen E Giwercman A Keiding N Skakkebaek NE. Evidence for Decreasing Quality of Semen During Past 50 Years. Br Med J (1992) 305(6854):609–13. doi: 10.1136/bmj.305.6854.609 PMC1883354 1393072

[4] Wingfield JC Sapolsky RM. Reproduction and Resistance to Stress: When and How. J Neuroendoc (2003) 15(8):711–24. doi: 10.1046/j.1365-2826.2003.01033.x 12834431

[5] Hansen PJ. Effects of Heat Stress on Mammalian Reproduction. Philos Trans R Soc B: Biol Sci (2009) 364(1534):3341–50. doi: 10.1098/rstb.2009.0131 PMC2781849 19833646

[6] Agarwal A Gupta S Sharma RK. Role of Oxidative Stress in Female Reproduction. Reprod Biol Endocrinol (2005) 3(1):28. doi: 10.1186/1477-7827-3-28 16018814 PMC1215514

[7] Alharbi OML Basheer AA Khattab RA Ali I. Health and Environmental Effects of Persistent Organic Pollutants. J Mol Liq (2018) 263:442–53. doi: 10.1016/j.molliq.2018.05.029

[8] Lopez-Rodriguez D Franssen D Bakker J Lomniczi A Parent A-S. Cellular and Molecular Features of Edc Exposure: Consequences for the Gnrh Network. Nat Rev Endocrinol (2021) 17(2):83–96. doi: 10.1038/s41574-020-00436-3 33288917

[9] Hedger MP. Immunophysiology and Pathology of Inflammation in the Testis and Epididymis. J Androl (2011) 32(6):625–40. doi: 10.2164/jandrol.111.012989 PMC7166903 21764900

[10] Leisegang K Henkel R Agarwal A. Obesity and Metabolic Syndrome Associated With Systemic Inflammation and the Impact on the Male Reproductive System. Am J Reprod Immunol (2019) 82(5):e13178. doi: 10.1111/aji.13178 31373727

[11] Hedger MP. Toll-Like Receptors and Signalling in Spermatogenesis and Testicular Responses to Inflammation—a Perspective. J Reprod Immunol (2011) 88(2):130–41. doi: 10.1016/j.jri.2011.01.010 PMC7127151 21333360

[12] Bhattacharya K Sengupta P Dutta S Karkada IR. Obesity, Systemic Inflammation and Male Infertility. Chemical Biology Letters (2020) 7(2):92–8.

[13] Weinbauer GF Luetjens CM Simoni M Nieschlag E. Physiology of Testicular Function. In: Nieschlag E Behre HM Nieschlag S, editors. Andrology: Male Reproductive Health and Dysfunction. Berlin, Heidelberg: Springer Berlin Heidelberg (2010). 11–59.

[14] Band DA Edelmann RJ Avery S Brinsden PR. Correlates of Psychological Distress in Relation to Male Infertility. Br J Health Psychol (1998) 3(3):245–56. doi: 10.1111/j.2044-8287.1998.tb00571.x

[15] Yang B Zhang J Qi Y Wang P Jiang R Li H. Assessment on Occurrences of Depression and Anxiety and Associated Risk Factors in the Infertile Chinese Men. Am J Men's Health (2017) 11(3):767–74. doi: 10.1177/1557988317695901 PMC5675225 28413943

[16] Lian F Shah A Mueller B Welliver C. Psychological Perspectives in the Patient With Chronic Orchialgia. Trans Androl Urol (2017) 6(Suppl 1):S14–S19. doi: 10.21037/tau.2017.03.91 PMC5503917 28725613

[17] Vellani E Colasante A Mamazza L Minasi MG Greco E Bevilacqua A. Association of State and Trait Anxiety to Semen Quality of in Vitro Fertilization Patients: A Controlled Study. Fertil Steril (2013) 99(6):1565–72.e2. doi: 10.1016/j.fertnstert.2013.01.098 23414918

[18] Nordkap L Jensen TK Hansen ÅM Lassen TH Bang AK Joensen UN. Psychological Stress and Testicular Function: A Cross-Sectional Study Of 1,215 Danish Men. Fertil Steril (2016) 105(1):174–87.e2. doi: 10.1016/j.fertnstert.2015.09.016 26477499

[19] Nordkap L Priskorn L Bräuner EV Marie Hansen Å Kirstine Bang A Holmboe SA. Impact of Psychological Stress Measured in Three Different Scales on Testis Function: A Cross-Sectional Study of 1362 Young Men. Andrology (2020) 8(6):1674–86. doi: 10.1111/andr.12835 32621382

[20] Bräuner EV Nordkap L Priskorn L Hansen ÅM Bang AK Holmboe SA. Psychological Stress, Stressful Life Events, Male Factor Infertility, And Testicular Function: A Cross-Sectional Study. Fertil Steril (2020) 113(4):865–75. doi: 10.1016/j.fertnstert.2019.12.013 32164925

[21] Fukuda M Fukuda K Shimizu T Yomura W Shimizu S. Kobe Earthquake and Reduced Sperm Motility. Hum Reprod (1996) 11(6):1244–6. doi: 10.1093/oxfordjournals.humrep.a019365 8671433

[22] Chen XM Chen SM Yue HX Lin L Wu YB Liu B. Semen Quality in Adult Male Survivors 5 Years After the 2008 Wenchuan Earthquake. Andrologia (2016) 48(10):1274–80. doi: 10.1111/and.12573 27135420

[23] Singh AK Jiang Y. How Does Peripheral Lipopolysaccharide Induce Gene Expression in the Brain of Rats? Toxicology (2004) 201(1):197–207. doi: 10.1016/j.tox.2004.04.015 15297033

[24] Jangula A Murphy EJ. Lipopolysaccharide-Induced Blood Brain Barrier Permeability Is Enhanced by Alpha-Synuclein Expression. Neurosci Lett (2013) 551:23–7. doi: 10.1016/j.neulet.2013.06.058 PMC3799901 23876253

[25] Fujii Y Yokochi T Nakashima I Asai J Kato N. A New Mouse Model for Autoimmune Orchitis. Med Microbiol Immunol (1991) 180(1):1–14. doi: 10.1007/BF00191695 2056961

[26] Lu K Lai KP Stoeger T Ji S Lin Z Lin X. Detrimental Effects of Microplastic Exposure on Normal and Asthmatic Pulmonary Physiology. J Hazard Mater (2021) 416:126069. doi: 10.1016/j.jhazmat.2021.126069 34492895

[27] Dym M Fawcett DW. The Blood-Testis Barrier in the Rat and the Physiological Compartmentation of the Seminiferous Epithelium. Biol Reprod (1970) 3(3):308–26. doi: 10.1093/biolreprod/3.3.308 4108372

[28] Nargund VH. Effects of Psychological Stress on Male Fertility. Nat Rev Urol (2015) 12(7):373–82. doi: 10.1038/nrurol.2015.112 26057063

[29] Tovote P Fadok JP Lüthi A. Neuronal Circuits for Fear and Anxiety. Nat Rev Neurosci (2015) 16(6):317–31. doi: 10.1038/nrn3945 25991441

[30] Rival C Theas MS Guazzone VA Lustig L. Interleukin-6 and Il-6 Receptor Cell Expression in Testis of Rats With Autoimmune Orchitis. J Reprod Immunol (2006) 70(1):43–58. doi: 10.1016/j.jri.2005.10.006 16458979

[31] Guazzone VA Rival C Denduchis B Lustig L. Monocyte Chemoattractant Protein-1 (Mcp-1/Ccl2) in Experimental Autoimmune Orchitis. J Reprod Immunol (2003) 60(2):143–57. doi: 10.1016/j.jri.2003.08.001 14638441

[32] Allen JA Diemer T Janus P Hales KH Hales DB. Bacterial Endotoxin Lipopolysaccharide and Reactive Oxygen Species Inhibit Leydig Cell Steroidogenesis *Via* Perturbation of Mitochondria. Endocrine (2004) 25(3):265–75. doi: 10.1385/ENDO:25:3:265 15758255

[33] Cudicini C Lejeune H Gomez E Bosmans E Ballet F Saez J. Human Leydig Cells and Sertoli Cells Are Producers of Interleukins-1 and -6. J Clin Endocrinol Metab (1997) 82(5):1426–33. doi: 10.1210/jcem.82.5.3938 9141528

[34] Riccioli A Starace D Galli R Fuso A Scarpa S Palombi F. Sertoli Cells Initiate Testicular Innate Immune Responses Through Tlr Activation. J Immunol (2006) 177(10):7122–30. doi: 10.4049/jimmunol.177.10.7122 17082629

[35] Feng R Adeniran SO Huang F Li Y Ma M Zheng P. The Ameliorative Effect of Melatonin on Lps-Induced Sertoli Cells Inflammatory and Tight Junctions Damage *Via* Suppression of the Tlr4/Myd88/Nf-Kb Signaling Pathway in Newborn Calf. Theriogenology (2022) 179:103–16. doi: 10.1016/j.theriogenology.2021.11.020 34871925

[36] Wong C-H Mruk DD Lui W-Y Cheng CY. Regulation of Blood-Testis Barrier Dynamics: An *In Vivo* Study. J Cell Sci (2004) 117(5):783–98. doi: 10.1242/jcs.00900 14734653

[37] Cheng CY Mruk DD. A Local Autocrine Axis in the Testes That Regulates Spermatogenesis. Nat Rev Endocrinol (2010) 6(7):380–95. doi: 10.1038/nrendo.2010.71 PMC4080676 20571538

[38] Cai Y Liu T Fang F Shen S Xiong C. Involvement of Icam-1 in Impaired Spermatogenesis After Busulfan Treatment in Mice. Andrologia (2016) 48(1):37–44. doi: 10.1111/and.12414 25808259

[39] Cheng CY Mruk DD. The Blood-Testis Barrier and Its Implications for Male Contraception. Pharmacol Rev (2012) 64(1):16–64. doi: 10.1124/pr.110.002790 22039149 PMC3250082

[40] BræNdstrur O Jensen L Werdelin O. Sertoli Cells, But Not Tumor Cells, of Seminoma in Situ Express Icam-1. APMIS (1996) 104(7-8):817–22. doi: 10.1111/j.1699-0463.1996.tb04947.x 8982245

[41] Mruk DD Cheng CY. The Mammalian Blood-Testis Barrier: Its Biology and Regulation. Endocr Rev (2015) 36(5):564–91. doi: 10.1210/er.2014-1101 PMC4591527 26357922

[42] Li X-X Chen S-R Shen B Yang J-L Ji S-Y Wen Q. The Heat-Induced Reversible Change in the Blood-Testis Barrier (Btb) Is Regulated by the Androgen Receptor (Ar) *Via* the Partitioning-Defective Protein (Par) Polarity Complex in the Mouse. Biol Reprod (2013) 89(1):1–10. doi: 10.1095/biolreprod.113.109405 23759306

[43] Li MWM Xia W Mruk DD Wang CQF Yan HHN Siu MKY. Tumor Necrosis Factor A Reversibly Disrupts the Blood–Testis Barrier and Impairs Sertoli–Germ Cell Adhesion in the Seminiferous Epithelium of Adult Rat Testes. J Endocrinol (2006) 190(2):313. doi: 10.1677/joe.1.06781 16899565

[44] Gerber J Heinrich J Brehm R. Blood–Testis Barrier and Sertoli Cell Function: Lessons From Sccx43ko Mice. Reproduction (2016) 151(2):R15. doi: 10.1530/rep-15-0366 26556893

[45] Qin L Wu X Block ML Liu Y Breese GR Hong JS. Systemic Lps Causes Chronic Neuroinflammation and Progressive Neurodegeneration. Glia (2007) 55(5):453–62. doi: 10.1002/glia.20467 PMC2871685 17203472

[46] Noh H Jeon J Seo H. Systemic Injection of Lps Induces Region-Specific Neuroinflammation and Mitochondrial Dysfunction in Normal Mouse Brain. Neurochem Int (2014) 69:35–40. doi: 10.1016/j.neuint.2014.02.008 24607701

[47] Valero J Mastrella G Neiva I Sánchez S Malva JO. Long-Term Effects of an Acute and Systemic Administration of Lps on Adult Neurogenesis and Spatial Memory. Front Neurosci (2014) 8:83(83). doi: 10.3389/fnins.2014.00083 24795557 PMC4001049

[48] Zhan X Stamova B Sharp FR. Lipopolysaccharide Associates With Amyloid Plaques, Neurons and Oligodendrocytes in Alzheimer’s Disease Brain: A Review. Front Aging Neurosci (2018) 10:42(42). doi: 10.3389/fnagi.2018.00042 29520228 PMC5827158

[49] Lehnardt S Lachance C Patrizi S Lefebvre S Follett PL Jensen FE. The Toll-Like Receptor Tlr4 Is Necessary for Lipopolysaccharide-Induced Oligodendrocyte Injury in the Cns. J Neurosci (2002) 22(7):2478–86. doi: 10.1523/jneurosci.22-07-02478.2002 PMC6758325 11923412

[50] Krasovska V Doering LC. Regulation of Il-6 Secretion by Astrocytes *Via* Tlr4 in the Fragile X Mouse Model. Front Mol Neurosci (2018) 11:272. doi: 10.3389/fnmol.2018.00272 30123107 PMC6085486

[51] Rolls A Shechter R London A Ziv Y Ronen A Levy R. Toll-Like Receptors Modulate Adult Hippocampal Neurogenesis. Nat Cell Biol (2007) 9(9):1081–8. doi: 10.1038/ncb1629 17704767

[52] Agrawal V Jaiswal MK Jaiswal YK. Gonadal and Nongonadal Fshr and Lhr Dysfunction During Lipopolysaccharide Induced Failure of Blastocyst Implantation in Mouse. J Assist Reprod Genet (2012) 29(2):163–73. doi: 10.1007/s10815-011-9696-4 PMC3270139 22193751

[53] Ying S Qin J Dai Z An H Zhu H Chen R. Effects of Lps on the Secretion of Gonadotrophin Hormones and Expression of Genes in the Hypothalamus-Pituitary-Ovary (Hpg) Axis in Laying Yangzhou Geese. Animals (2020) 10(12):2259. doi: 10.3390/ani10122259 PMC7760895 33266293

[54] Miller SM Lonstein JS. Dopaminergic Projections to the Medial Preoptic Area of Postpartum Rats. Neuroscience (2009) 159(4):1384–96. doi: 10.1016/j.neuroscience.2009.01.060 PMC2888488 19409227

[55] Kohl J Babayan BM Rubinstein ND Autry AE Marin-Rodriguez B Kapoor V. Functional Circuit Architecture Underlying Parental Behaviour. Nature (2018) 556(7701):326–31. doi: 10.1038/s41586-018-0027-0 PMC5908752 29643503

[56] Ricardo JA Tongju Koh E. Anatomical Evidence of Direct Projections From the Nucleus of the Solitary Tract to the Hypothalamus, Amygdala, and Other Forebrain Structures in the Rat. Brain Res (1978) 153(1):1–26. doi: 10.1016/0006-8993(78)91125-3 679038

[57] Herman JP Prewitt CM-F Cullinan WE. Neuronal Circuit Regulation of the Hypothalamo-Pituitary-Adrenocortical Stress Axis. Crit Rev Neurobiol (1996) 10(3-4):371–94. doi: 10.1615/CritRevNeurobiol.v10.i3-4.50 8978987

[58] Caligioni CS Oliver C Jamur MC Franci CR. Presence of Oxytocin Receptors in the Gonadotrophin-Releasing Hormone (Gnrh) Neurones in Female Rats: A Possible Direct Action of Oxytocin on Gnrh Neurones. J Neuroendoc (2007) 19(6):439–48. doi: 10.1111/j.1365-2826.2007.01550.x 17504438

[59] McCall Jordan G Al-Hasani R Siuda Edward R Hong Daniel Y Norris Aaron J Ford Christopher P. Crh Engagement of the Locus Coeruleus Noradrenergic System Mediates Stress-Induced Anxiety. Neuron (2015) 87(3):605–20. doi: 10.1016/j.neuron.2015.07.002 PMC4529361 26212712

[60] Ota A Kaneko YS Mori K Nakashima A Nagatsu I Nagatsu T. Effect of Peripherally Administered Lipopolysaccharide (Lps) on Gtp Cyclohydrolase I, Tetrahydrobiopterin and Norepinephrine in the Locus Coeruleus in Mice. Stress (2007) 10(2):131–6. doi: 10.1080/10253890701350511 17514581

[61] Li L Feng X Zhou Z Zhang H Shi Q Lei Z. Stress Accelerates Defensive Responses to Looming in Mice and Involves a Locus Coeruleus-Superior Colliculus Projection. Curr Biol (2018) 28(6):859–71.e5. doi: 10.1016/j.cub.2018.02.005 29502952

[62] Williams LM. Precision Psychiatry: A Neural Circuit Taxonomy for Depression and Anxiety. Lancet Psychiatry (2016) 3(5):472–80. doi: 10.1016/S2215-0366(15)00579-9 PMC4922884 27150382

[63] Lüthi A Lüscher C. Pathological Circuit Function Underlying Addiction and Anxiety Disorders. Nat Neurosci (2014) 17(12):1635–43. doi: 10.1038/nn.3849 25402855

[64] Calhoon GG Tye KM. Resolving the Neural Circuits of Anxiety. Nat Neurosci (2015) 18(10):1394–404. doi: 10.1038/nn.4101 PMC7575249 26404714

[65] Huang S-H Liu W-Z Qin X Guo C-Y Xiong Q-C Wang Y. Association of Increased Amygdala Activity With Stress-Induced Anxiety But Not Social Avoidance Behavior in Mice. Neurosci Bull (2022) 38(1):16–28. doi: 10.1007/s12264-021-00762-0 34494228 PMC8782949

