# Supplementary figures and images for: LPS-Induced Systemic Inflammation Caused mPOA-FSH/LH Disturbance and Impaired Testicular Function
Source: Front Endocrinol (Lausanne). 2022 Jun 23;13:886085. doi: 10.3389/fendo.2022.886085 (PMC9259990; doi:10.3389/fendo.2022.886085)

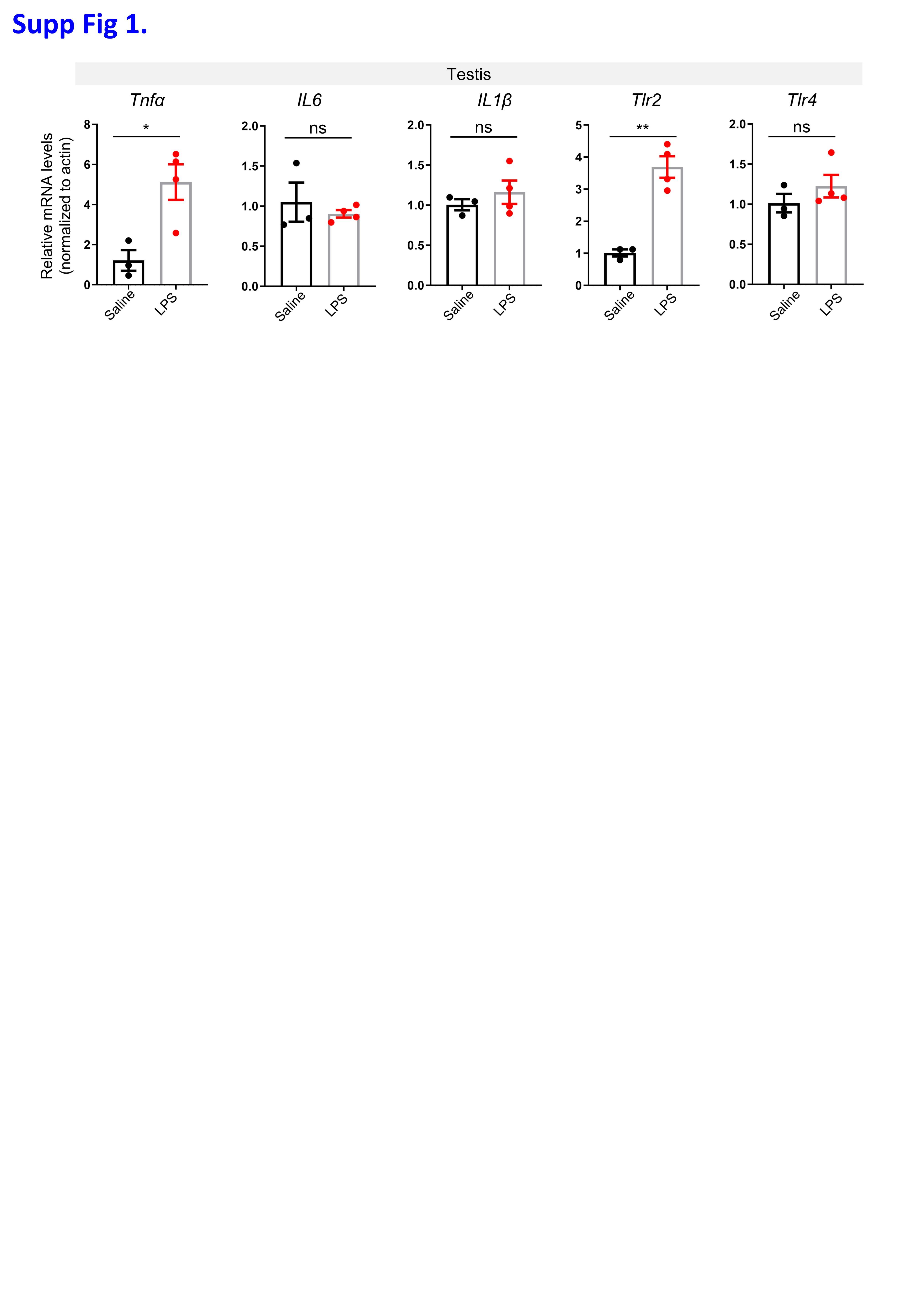

Supplement: Supplementary Figure 1 — LPS caused increase of inflammatory cytokines gene expression in the mouse testis. Relative mRNA levels normalized to actin showed significant increase in the levels of Tnfα and Tlr2 (data are presented as mean ± SEM, n = 3 Saline, n = 4 LPS; Student’s t test, ** p < 0.01, * p < 0.05, ns, p > 0.05). [file Image_1.jpg]

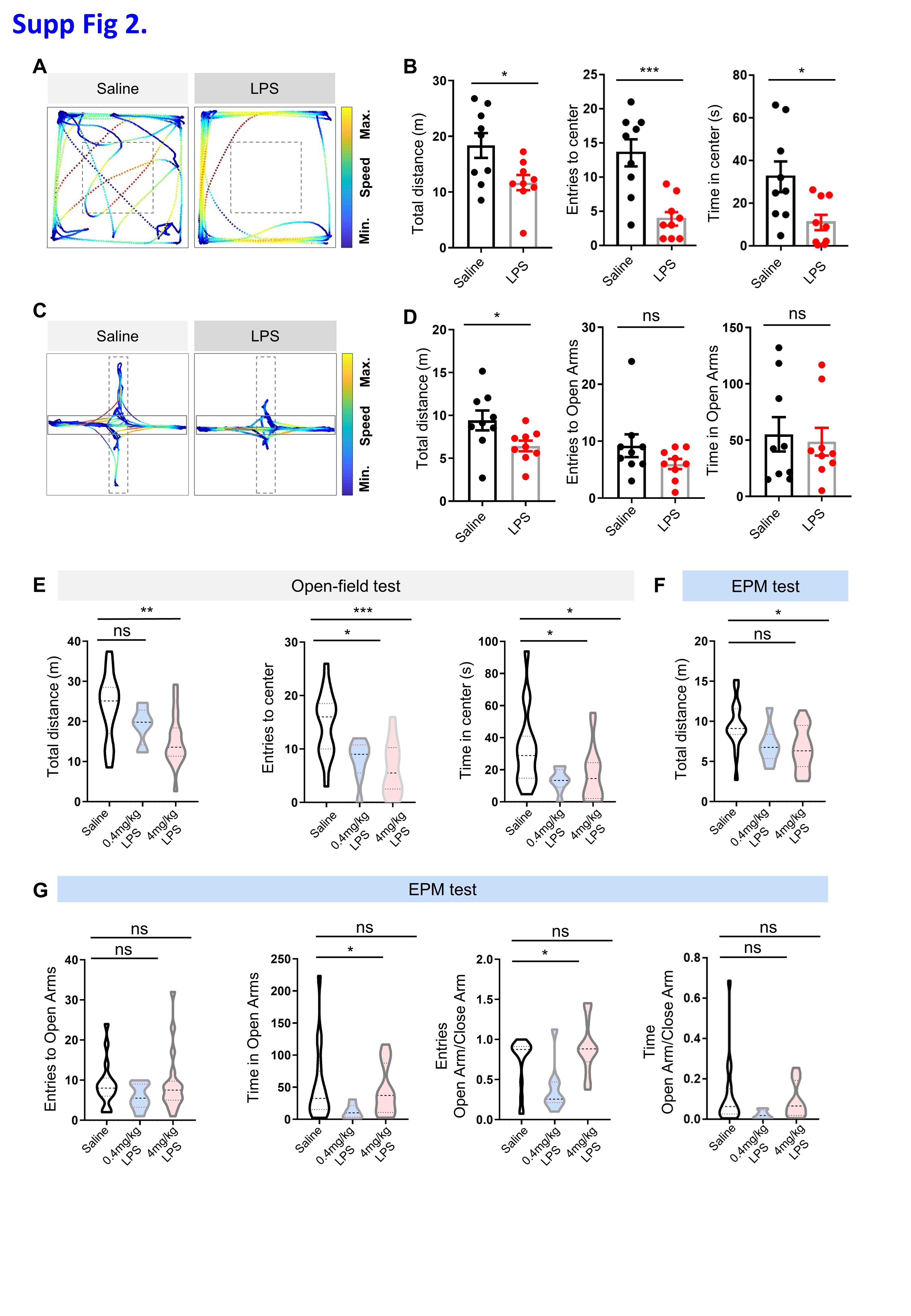

Supplement: Supplementary Figure 2 — LPS treated mice showed abnormal behaviors in open-field test and elevated plus maze test 48 hours after LPS treatment. (A) Example of OFT tracking traces of mice in the saline control and LPS groups. (B) LPS-treated mice had decreased total distance travelled, fewer entries to the center and less time in the center (data are presented as mean ± SEM, n = 9 Saline, n = 9 LPS; Student’s t test, ***p < 0.001, *p < 0.05). (C) Example of EPM tracking traces of mice in the saline control and LPS groups. (D) LPS-treated mice had decreased total distance travelled (data are presented as mean ± SEM, n = 9 Saline, n = 9 LPS; Student’s t test, *p < 0.05, ns, p > 0.05). (E) In open-field test, LPS at 4mg/kg caused decreases in Total distance travelled, Entries to center and Time in center, LPS at 0.4mg/kg caused decreases in Total distance travelled, Entries to center and Time in center (data are presented as Violin plot, n = 17 Saline, n = 8 0.4mg/kg LPS, n = 18 4mg/kg LPS; Kruskal-Wallis with Dunn post-hoc test, ***p < 0.001, **p < 0.01, *p < 0.05, ns, p > 0.05). (F–G) In the elevated plus maze test, 4 mg/kg LPS caused decrease in total distance travelled, 0.4mg/kg LPS caused no significant difference in Total distance travelled and a significant decrease in the ratio of Entries to Open Arms and Entries to Close Arms (data are presented as Violin plot, n = 17 Saline, n = 8, 0.4mg/kg LPS, n = 18 for 4mg/kg LPS; Kruskal-Wallis with Dunn post-hoc test, *p < 0.05, ns, p > 0.05). [file Image_2.jpg]

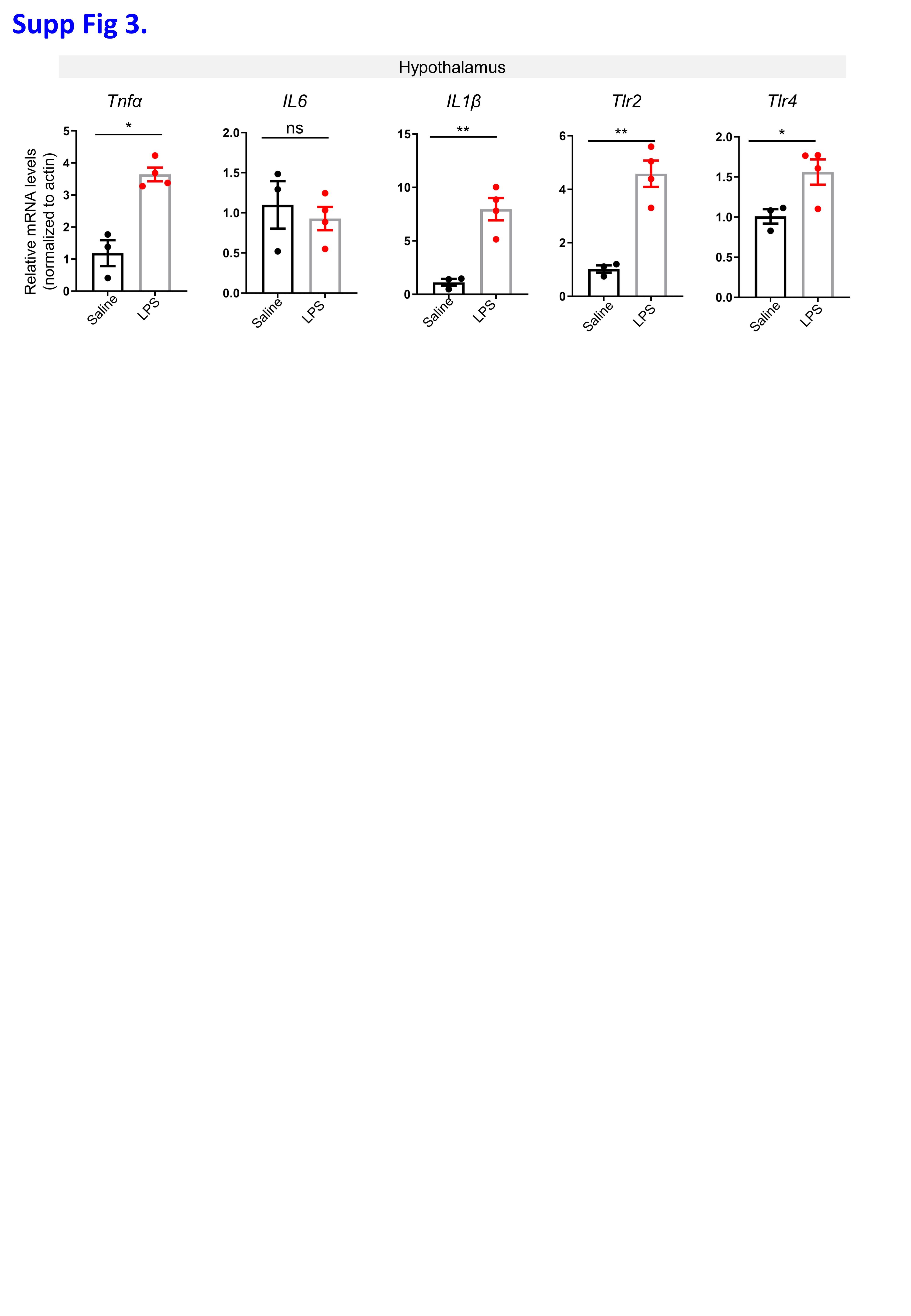

Supplement: Supplementary Figure 3 — LPS caused increase of inflammatory cytokines gene expression in the mouse hypothalamus. Relative mRNA levels normalized to actin showed significant increase in the levels of Tnfα, IL1β, Tlr2 and Tlr4 (data are presented as mean ± SEM, n = 3 Saline, n = 4 LPS; Student’s t test, ** p < 0.01, * p < 0.05, ns, p > 0.05). [file Image_3.jpg]
